# Assessment of the knowledge of graphical symbols labelled on malaria rapid diagnostic tests in four international settings

**DOI:** 10.1186/1475-2875-10-331

**Published:** 2011-11-02

**Authors:** Veerle Hermans, Lianet Monzote, Björn Van den Sande, Pierre Mukadi, Thai Sopheak, Philippe Gillet, Jan Jacobs

**Affiliations:** 1 Department of Clinical Sciences, Institute of Tropical Medicine, Unit of Tropical Laboratory Medicine, Nationalestraat 155, B 2000 Antwerp, Belgium; 2 Institute of Tropical Medicine Pedro Kourí, La Habana, Cuba; 3 Quality Assurance, Institute of Tropical Medicine, Antwerp, Belgium; 4 Institut National de Recherche Biomédicale, Kinshasa, Democratic Republic of the Congo; 5 Sihanouk Hospital Center of HOPE, Phnom Penh, Cambodia; 6 Department of Medical Microbiology, Faculty of Medicine and Health Sciences, University of Maastricht, The Netherlands

**Keywords:** Graphical symbols, *in vitro *diagnostics, ISO 15223, malaria rapid diagnostic tests

## Abstract

**Background:**

Graphical symbols on *in vitro *diagnostics (IVD symbols) replace the need for text in different languages and are used on malaria rapid diagnostic tests (RDTs) marketed worldwide. The present study assessed the comprehension of IVD symbols labelled on malaria RDT kits among laboratory staff in four different countries.

**Methods:**

Participants (n = 293) in Belgium (n = 96), the Democratic Republic of the Congo (DRC, n = 87), Cambodia (n = 59) and Cuba (n = 51) were presented with an anonymous questionnaire with IVD symbols extracted from ISO 15223 and EN 980 presented as stand-alone symbols (n = 18) and in context (affixed on RDT packages, n = 16). Responses were open-ended and scored for correctness by local professionals.

**Results:**

Presented as stand-alone, three and five IVD symbols were correctly scored for comprehension by 67% and 50% of participants; when contextually presented, five and seven symbols reached the 67% and 50% correct score respectively. 'Batch code' scored best (correctly scored by 71.3% of participants when presented as stand-alone), 'Authorized representative in the European Community' scored worst (1.4% correct). Another six IVD symbols were scored correctly by less than 10% of participants: 'Do not reuse', '*In vitro *diagnostic medical device', 'Sufficient for', 'Date of manufacture', 'Authorised representative in EC', and 'Do not use if package is damaged'. Participants in Belgium and Cuba both scored six symbols above the 67% criterion, participants from DRC and Cambodia scored only two and one symbols above this criterion. Low correct scores were observed for safety-related IVD symbols, such as for 'Biological Risk' (42.7%) and 'Do not reuse' (10.9%).

**Conclusion:**

Comprehension of IVD symbols on RDTs among laboratory staff in four international settings was unsatisfactory. Administrative and outreach procedures should be undertaken to assure their acquaintance by end-users.

## Background

### Graphical symbols on *in vitro *diagnostics

**Figure 1:**
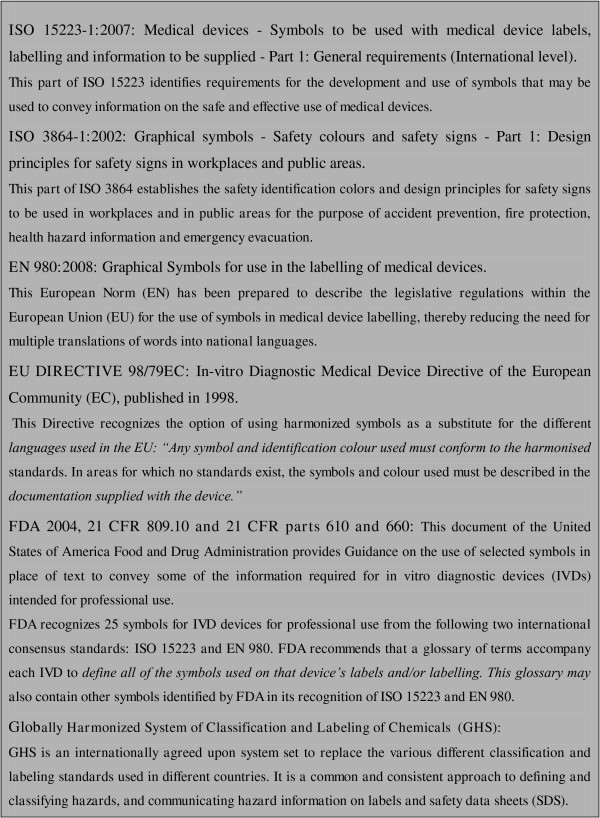
**International standard regulations that provide guidelines on the use of symbols on IVDs and medical devices**.

To achieve international market compliance, medical devices and *in vitro *diagnostics (IVDs) must display technical and safety information on their packaging. For that purpose, graphical symbols as published in the International Organization of Standardization (ISO 15223) and the European Norm EN 980 may be used. Both the European Union and the US Food and Drug Administration (through directive 98/79/EC and FDR 21 809.10 and 21 respectively) recommend the use of graphical symbols on medical devices and *in vitro *diagnostic devices, further referred to as 'IVD symbols' (Figure 1). Apart from obviating the need for supplying information in different languages, graphical symbols have advantages over full text such as high visual impact and noticeability [1].

In a previous study, IVD symbols were noted on the labels of all 40 boxes of malaria rapid diagnostic kits (RDTs) [2]. Although it might be assumed that laboratory staff all over the world would be familiar with the meaning of IVD symbols, this assumption has to be confirmed. The present study was done as part of a network programme on laboratory quality management: as a preparation of dedicated training on IVD symbols, the comprehension of diagnostic symbols figuring on RDT kits was assessed among laboratory staff in four different settings.

## Methods

### Study sites and ethical considerations

The survey was conducted at institutions in four different countries: (i) Institute of Tropical Medicine (ITM), Antwerp, Belgium, (ii) Institut National de Recherche Biomédicale (INRB), Kinshasa, Democratic Republic of the Congo (DRC), (iii) Sihanouk Hospital Centre of Hope (SHCH), Phnom Penh, Cambodia, (iv) Institute of Tropical Medicine Pedro Kouri (IPK), Havana, Cuba. The study consisted of an on-site survey during which an anonymous questionnaire was presented. The study was approved by the Institutional Review Board (IRB) of ITM, Antwerp, and by the Ethical Committee of Antwerp University, Belgium.

### Period, participants and recruitment

The survey was conducted between April 2009 and September 2009. The targeted study participants were laboratory health care workers (HCW) who worked regularly with IVDs in diagnostic laboratories: doctors, pharmacists, biologists, chemists, laboratory technicians and students in biomedical science and medicine. Participants were addressed by the local collaborator at each study site during breaks in training sessions or meetings on unrelated topics.

### Questionnaire

**Figure 2:**
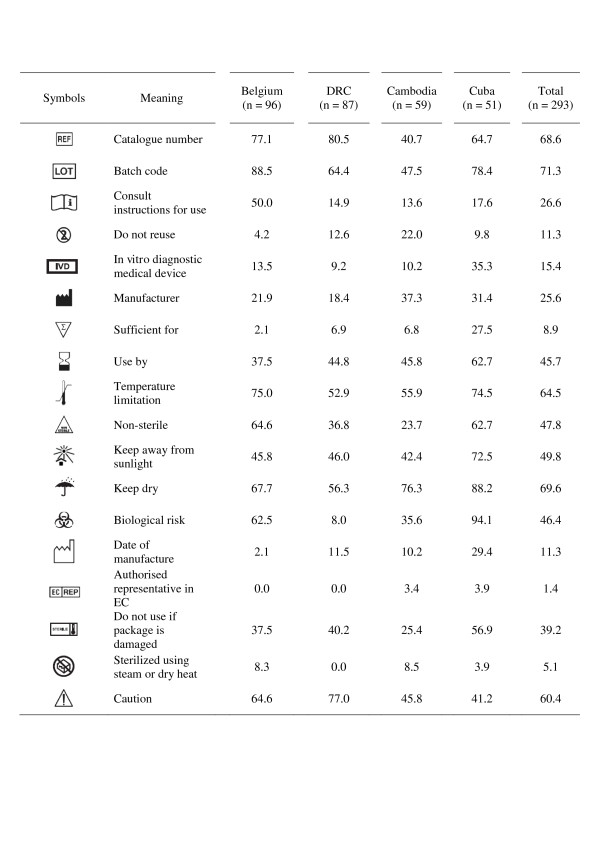
**Graphical symbols used in this survey, their meaning and percentage of correct answers by participants from the different settings, when assessed as stand-alone symbols**.

The questionnaire was available in four languages (Dutch, English, French and Spanish), which had been back-translated by local collaborators. It was preceded by an information letter explaining the purpose of the study and asking for consent. Next, written instructions were given. Eighteen IVD symbols were included: all were IVD symbols used on RDT kits and extracted from ISO 15223 and EN 980 [3,4]; they were presented in black-and-white prints (Figure 2). The questionnaire consisted of two parts: the first part presented the IVD symbols as stand-alone symbols and the second part presented 16 of them in context, *i.e*. as displayed on a colour photograph of a malaria RDT kit package. The questionnaire was open-ended: participants were asked to write down in their own words the presumed meaning, according to ISO 9186 [1,3]. Questionnaires were collected immediately after completion.

### Sample sizes, end points, data registration and analysis

In line with other studies on symbol comprehension, a target of 50 to 100 participants for each setting was set [1,5]. Answers were compared to the correct meaning of each symbol as described in ISO 15223 and EN 980 and interpreted as correct or incorrect. A correct answer meant that the meaning was identical or fully consistent with the reference. Blanks and answers like 'I don't know' were assigned to the category 'incorrect'. Interpretations were done in consensus with the local collaborator at each site. For definition of an acceptable score, the criterion used by Liu and co-workers evaluating IVD symbols in intensive care units was used, i.e. ≥ 67% of surveyed participants answering correctly based on the ISO 3864 version 1984 [1,6]. In addition, a second, more permissive threshold of 50%, correct score was used, in line with Kassam and co-workers assessing pictographic instructions for medications [7]. Data were collected on A4 paper folders and subsequently labelled with a single identifier. They were recorded in a Microsoft Excel spreadsheet. Proportions were tested with the Pearson's chi-square (χ^2^) test. A p-value < 0.05 was considered to be significant.

## Results

### Participants

A total of 293 HCW from four settings participated: Belgium: n = 96, DRC: n = 87, Cambodia: n = 59 and Cuba: n = 51. The male-to-female ratio was 1:0.74. Of the 293 participants, a Bachelor's degree was held by 49.1%, 35.5% held Master degrees; the remaining participants were students. Professional experience in years was highest among the participants from Cuba (median 18.5 years, range 2-26), followed by participants from Belgium (median 9.5 years, range 0.5-37), Cambodia (median 8.0 years, range 0.5-25) and DRC (median 4.5 years, range 0-35).

### Comprehension of IVD symbols as stand-alone symbols

For all 293 participants combined, three and five out of 18 symbols reached the 67% and 50% scores (Figure 2). 'Batch code' scored best (correctly scored by 71.3% of participants) and the lowest score was noted for 'Authorized representative in EC' (1.4%). None of the IVD symbols reached the 67% score in all four settings but two reached the 50% score ('Temperature limitation' and 'Keep dry'). Participants of Belgium and Cuba both scored six symbols above the 67% ISO 3864 criterion, participants from DRC and Cambodia scored two and one symbol above this criterion respectively. The most striking difference between the settings was noted for the 'Biological risk' symbol, which was scored correctly by 94.1% and 8.0% of participants from Cuba and DRC respectively (p < 0.001). Another six IVD symbols were scored correctly by less than 10% of participants in all four settings: 'Do not reuse', '*In vitro *diagnostic medical device', 'Sufficient for', 'Date of manufacture', 'Authorised representative in EC', and 'Do not use if package is damaged'.

### Comprehension of symbols when presented in context

**Figure 3:**
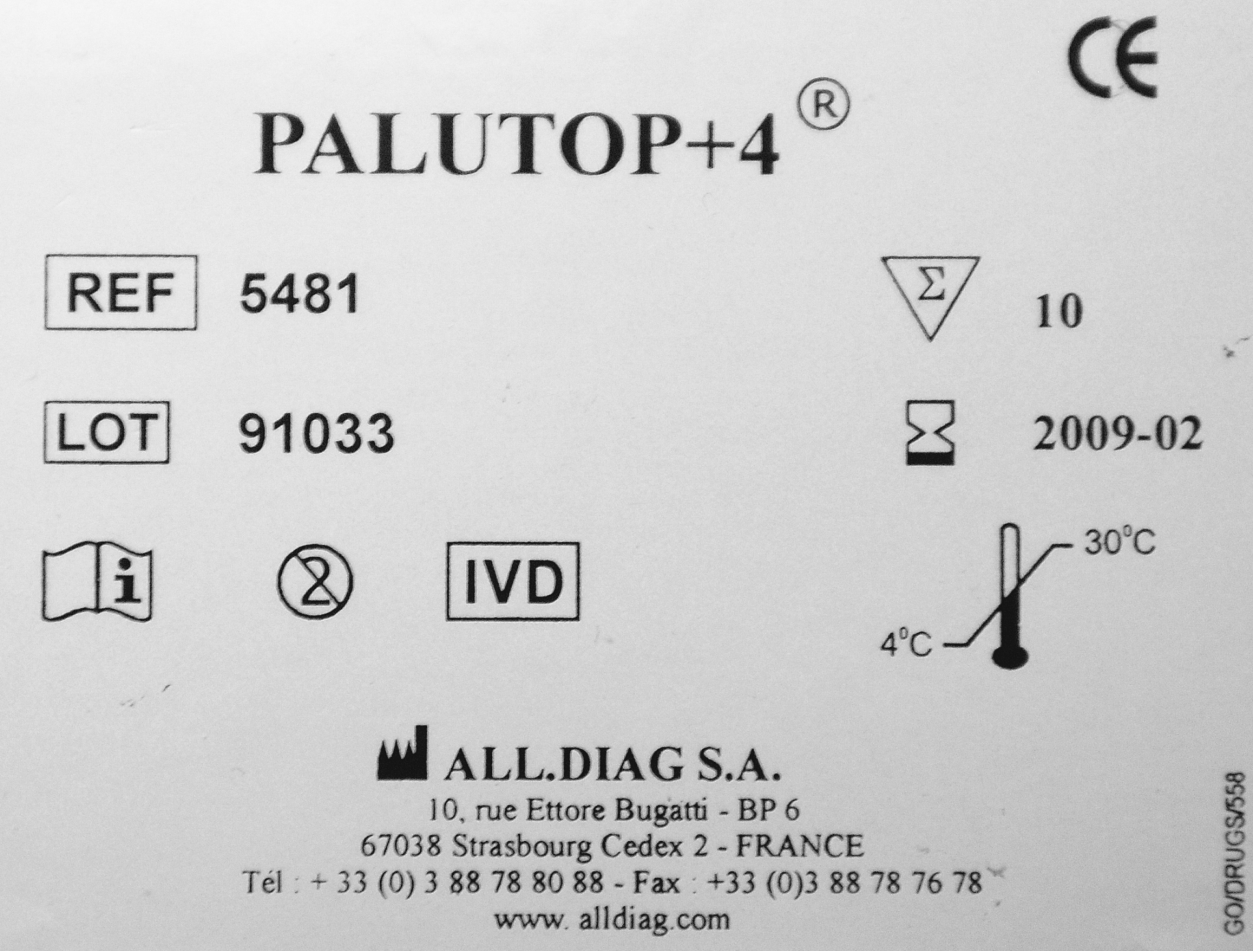
**A package of malaria RDT *Palutop*^® ^*+4***. This picture was used to present 10 IVD symbols in context.

**Figure 4:**
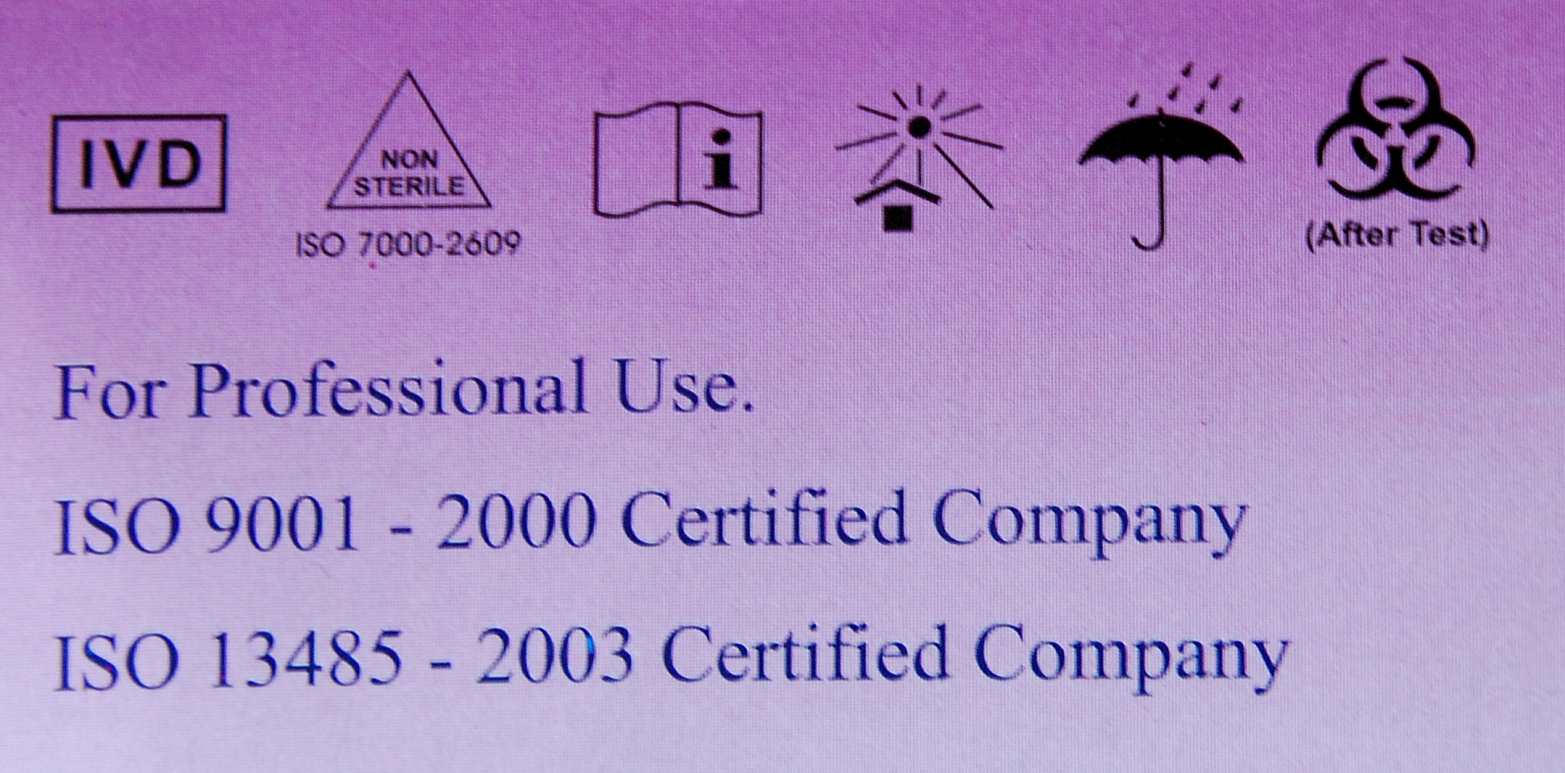
**A package of malaria RDT First Response^® ^malaria antigen test" This picture was used to present four additional IVD symbols in context ('Non-sterile', 'Keep away from sunlight', 'Keep dry' and 'Biological risk')**.

**Figure 5:**
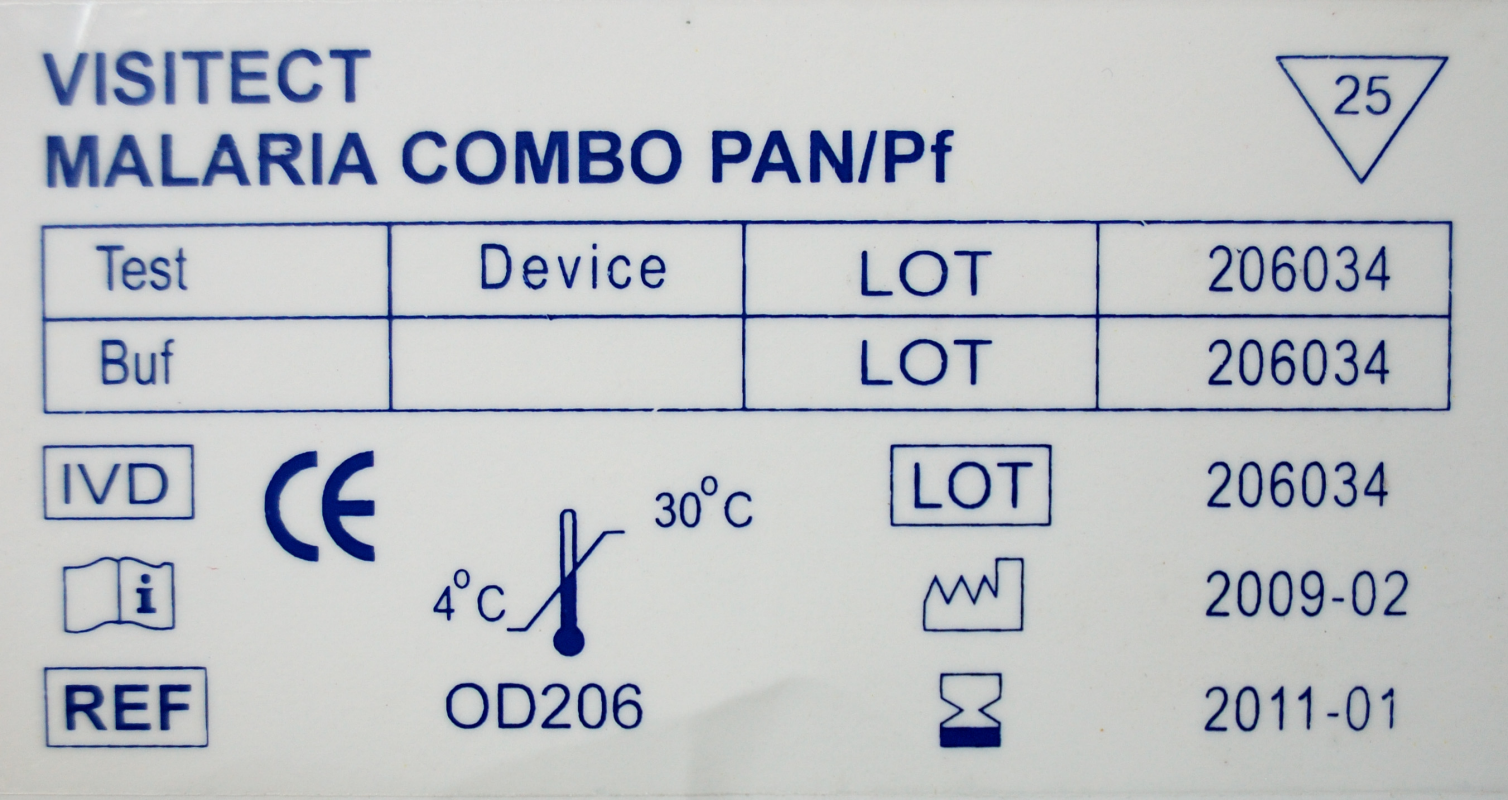
**A package of malaria RDT Visitec malaria combo pan/Pf**. This picture was used to present the IVD symbol 'Date of manufacture' in context. NB: the symbol used for 'Sufficient for' is not required by EN 980: the inverted triangle should contain the '∑' character and affix the number beneath.

**Figure 6:**
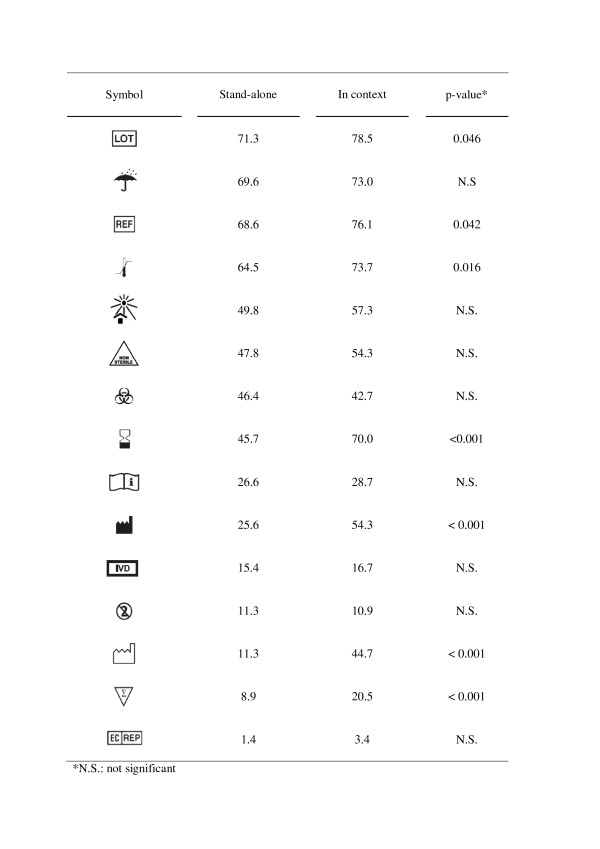
**Percentages of overall correct scores for each symbol when presented as stand-alone versus in context for 293 participants**. Symbols are ranked from highest to lowest score when presented as stand-alone.

Sixteen symbols among those shown in the first part (stand-alone symbols) were presented in context by showing pictures of malaria RDT packages (Figures 3, 4 and 5). Seven of these 16 symbols that were presented in context were correctly identified significantly more frequently compared to their scores as stand-alone symbols (Figure 6): 'Catalogue number', 'Batch code', 'Temperature limitation', 'Use by', 'Manufacturer', 'Date of manufacture' and 'Sufficient for', with overall correct scores of respectively 78.5%, 76.1%, 70.0%, 54.3%, 44.7% and 20.5%. No increase at all was noted for the low-scoring 'Consult instructions for use', '*In vitro *diagnostic medical device', 'Do not reuse' and 'Authorised representative in EC'. There were no differences in improved identification between the four study settings. Overall, five and seven diagnostic symbols reached the 67% ISO 3864 and the 50% criteria respectively.

## Discussion

### The use of graphical symbols on IVDs is encouraged by EU and FDA

To be released on international markets, RDTs like other IVDs are required to display product information on their packaging, including information about manufacturer, storage temperature, manufacture and expiration dates and several instructions for use [2]. To avoid the need for translation of this information in different languages and to comply with space limitations, manufacturers use IVD symbols that convey the required information [8]. Both EU and FDA regulations recognize and authorize the use of these IVD symbols. In the EU, the original motivation for stimulating the use of graphic symbols was to obviate the need for translating the information in all national languages of the community, but other advantages such as noticeability, standardization and lower risks of labelling errors are now recognized [3,9].

### Limitations of the present study

The present study has some limitations. For instance, although they were instructed not to do so, participants filling out the second part (symbols in context) were theoretically able to go back to the first part (stand-alone symbols) and make corrections. However, the significant differences in scores between both categories indicated that this had not occurred in practice. Further, despite complying with the professional languages of the target audience, writing in English or French proved difficult for some participants in Cambodia and DRC, which in turn led to occasional difficulty in scoring responses. The option of multiple-choice questions would have avoided possible errors in interpretation. However, the so-called open-ended comprehension method is recommended for surveys of symbol comprehension, such as for IVD and safety symbols or icons in consumer medical information [1,10-12], and multiple-choice questions would have suffered from interferences of recall and suggestion [13]. In addition, reliability in the present study was assured by interpreting the answers in collaboration with a local collaborator who corrected for linguistic factors. Finally, participants' familiarity with RDTs was not registered, although the majority of them had little experience. Among the four settings, malaria is only endemic in DRC, but malaria RDTs were not yet released at the time of the survey. Nevertheless, all participants were actual end-users of IVDs (such as HIV RDTs) and hence supposed to be exposed to IVD symbols. Finally, it should be noted that the present study did not assess the factual consequences of non-comprehension of symbols in terms of practical handling and diagnostic accuracy.

### The comprehension of IVD symbols on RDTs is poor

The present study showed that laboratory staff in different international settings scored poorly at comprehension of IVD symbols displayed on RDTs. This is in contrast to the general perception of regulatory authorities: FDA refers to internal validation processes to claim understanding of the symbols by laboratory users of various educational backgrounds, providing acquaintance through information leaflets and training. Likewise, EN 980:2008 describes the IVD symbols as self-evident to healthcare professionals with no need for further explanation (with the exception of the symbol 'Do not use when package is damaged') [3,9]. Of most concern are the low scores for the IVD symbols related to safety, e.g. 'Biological risk', 'Do not reuse' and 'Consult instructions before use', even when presented in context, since IVD symbols are considered an important element in risk reduction [3]. Despite these claims, it is clear that actual comprehension of the IVD symbols does not reach the intended level, which suggest to review the comprehension validation procedures.

Most graphical symbols in use in community or professional life pertain to transportation, industry or trade [14] and few evaluations of the comprehension of graphical symbols among end-users have been conducted, particularly in non-industrialized countries. When performed, comprehension studies focused on hazard or chemical symbols and addressed industry, agriculture of trade workers [5,15]. Only a single study assessed the comprehension of symbols among HCW in Germany and China: among the surveyed symbols (displayed on electrical and medical equipment,), there were two IVD symbols, 'Date of manufacture' and 'Do not reuse'. These two symbols scored poorly (correct by less than 50% of participants) both in Germany and China.

### Comprehension of symbols: pictorial and abstract designs

Graphical symbols are either pictorial/iconic (i.e. presenting familiar objects from daily life or alphanumeric data) or abstract (displaying an arbitrary figure). In the present survey, pure pictorial symbols, such as the umbrella of the 'Keep dry' symbol, generally scored better compared to abstract symbols, which scored lower even when presented in context and with dates or numbers referring to their meaning. The symbols 'Date of manufacture' and 'Sufficient for' did not reach the 50% correct score. Similar findings were observed for chemical hazard symbols surveyed among agricultural and industrial workers in Zambia: flame-like symbols were better understood than the St Andrew's cross [15]. On the other hand, pictorial symbols may entail confounders: i.e. the 'Do not reuse' symbol was explained as 'Do not give to children under two years old' by several participants in the present survey.

### Comprehension of symbols: design, acquaintance, education and culture

Acquaintance and familiarity with graphical symbols are other factors favouring comprehension. For instance, the symbol 'Biological risk'-an abstract symbol-was best scored by the participants from Cuba, who had participated to a training in bio-safety matters shortly before the survey. Training and education are essential tools in the comprehension of chemical and safety symbols [16]. Likewise, the symbol 'Consult instructions before use' was scored best by the participants in Belgium, probably by a familiarity with other types of consumables and equipment marketed in the EU and affixing these symbols.

Finally, there is a possible role for cultural and educational factors. The present study was not designed to assess such factors and published literature provides little information about the relationship between cultural background and symbol comprehension. In a community setting in South Africa, locally designed pictograms for medicine instructions were better comprehended than internationally available pictograms [10], whereas a study of medicine instructions among immigrants of non-European descent in Canada showed that interpretation was related to educational level and visual literacy rather than to ethnic or demographic factors [7]. In the aforementioned study of symbols in intensive care units in Germany and China, no apparent cultural differences were identified but professional experience was identified as a factor influencing symbol comprehension [1].

### Improving the comprehension of IVD symbols

**Figure 7:**
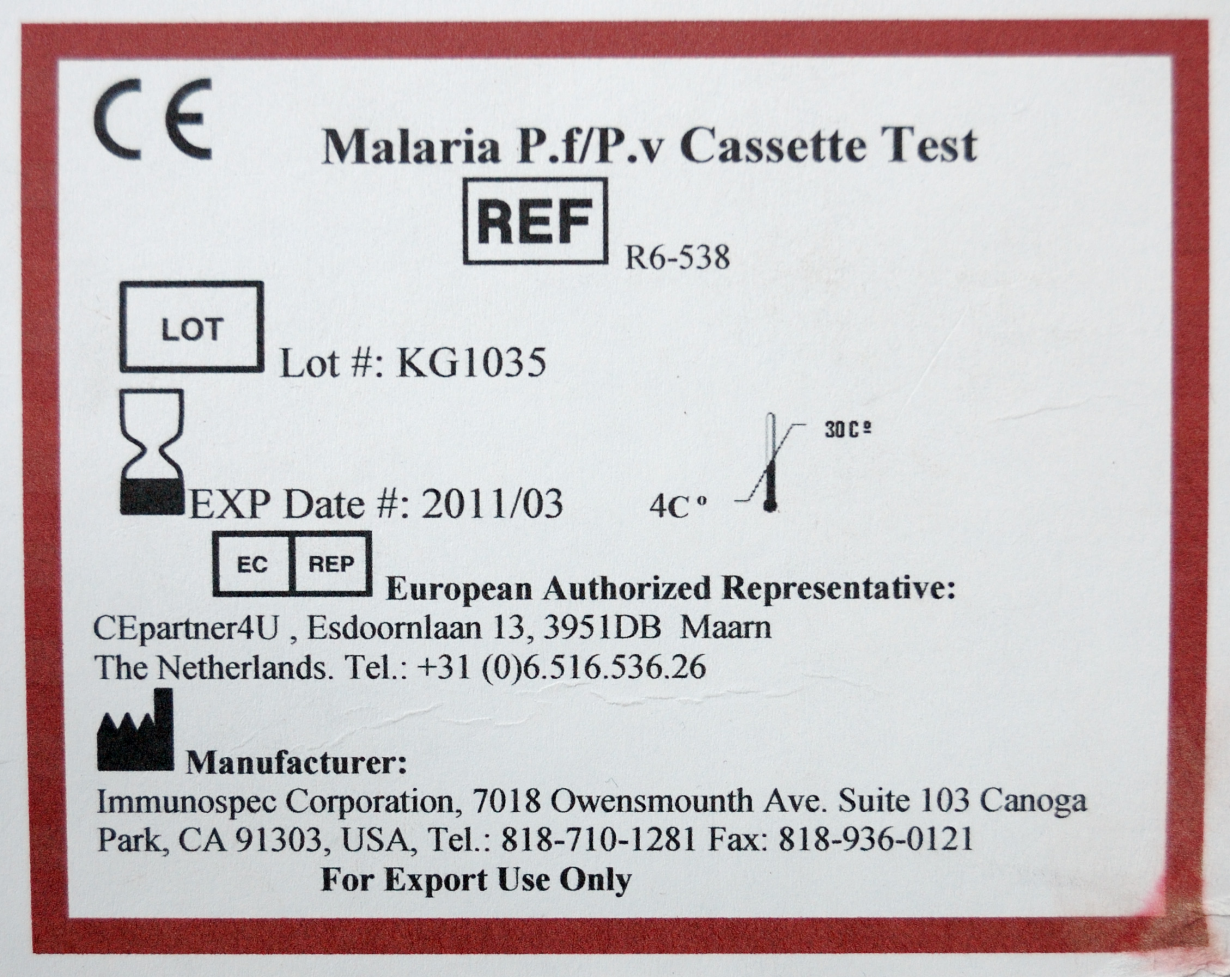
**A package of malaria RDT Immunospec malaria Pf/Pv cassette test, using the synergistic effect to show both pictorial aids and textual instructions**.

Studies on the information transfer of consumer medical information clearly showed the synergistic effect of pictorial aids and textual instructions: displaying text together with symbols improves attention, comprehension and recall of the message, as is practiced on some RDT packages (Figure 7) [10,17-20]. To increase acquaintance with the symbols, FDA recommends that IVD manufacturers add an explanatory glossary of the affixed IVD symbols [9]: this practice was recorded in 9/42 (21.4%) RDT brands assessed for quality of labelling and information sheets [2]. Another FDA recommendation is to organize educational outreach efforts by training, letters, posters and advertisements in professional journals and websites [9]. Finally, for the introduction of new symbols, the use of pictorial rather than abstract symbols should be considered.

## Conclusion

In conclusion, comprehension of IVD symbols on RDTs among laboratory staff in four international settings was unsatisfactory. Administrative and outreach procedures should be undertaken to explain the meaning of IVD symbols and to assure their acquaintance by end-users.

## List of abbreviations

EC: European Commission; EC-REP: European Authorized Representative; EN: European norm; EU: European Union; FDA: Food and Drug Administration; DRC: Democratic Republic of the Congo; HCW: health care worker; ISO: International Organization of Standardization; INRB: Institut National de Recherche Biomédicale; IPK: Institute of Tropical Medicine Pedro Kouri; IRB: Institutional Review Board; ITM: Institute of Tropical Medicine; IVD: *I*n *vitro *diagnostic; RDT(s): rapid diagnostic test(s); SHCH: Sihanouk Hospital Centre of Hope

## Competing interests

The authors declare that they have no competing interests.

## Authors' contributions

VH, BVDZ and JJ designed the study protocol, instructions and questionnaire. LM, PM and TS validated the questionnaire and scored the responses. PG and VH carried out the analysis. VH, LM, BVDZ and PG drafted the manuscript. All authors have read and approved the manuscript.
